# Paradoxical effects of lipolysis on the lipid oxidation in meat and meat products

**DOI:** 10.1016/j.fochx.2022.100317

**Published:** 2022-04-23

**Authors:** Nantawat Tatiyaborworntham, Fatih Oz, Mark P. Richards, Haizhou Wu

**Affiliations:** a Food Biotechnology Research Team, National Center for Genetic Engineering and Biotechnology (BIOTEC), 113 Thailand Science Park, Phahonyothin Road, Pathum Thani 12120, Thailand; b Department of Food Engineering, Faculty of Agriculture, Ataturk University, 25240 Erzurum, Turkey; c Department of Animal and Dairy Sciences, University of Wisconsin-Madison, Meat Science and Animal Biologics Discovery, 1933 Observatory Dr. Madison, WI 53706, United States; d Department of Biology and Biological Engineering-Food and Nutrition Science, Chalmers University of Technology, SE 412 96 Gothenburg, Sweden

**Keywords:** Lipolysis, Phospholipase, Lipase, Free fatty acid, Hemoglobin, Myoglobin, Phospholipid

## Abstract

Lipolysis in meat and meat products is a phenomenon involving hydrolysis of lipids, notably via enzymatic catalysis that takes place even *postmortem*. During refrigerated and frozen storage of meat, in particular fish, endogenous lipolytic enzymes actively degrade triacylglycerols and phospholipids resulting in accumulation of free fatty acids and other hydrolytic products. A classical conjecture suggests that lipolysis enhances lipid oxidation which is involved in quality deterioration of fresh meat and, to some degrees, flavor development of certain meat products. Recent studies (<5 years) have shown that under some circumstances, lipolysis of certain lipolytic enzymes can inhibit lipid oxidation in muscle models, which provides more insight in lipid oxidation mechanisms in muscle matrices as well as implies potential strategies for improving meat quality. This review will discuss such paradoxical effects and potential mechanisms of lipolysis on lipid oxidation in meat and meat products.

## Highlights

• Lipolysis and lipid oxidation occur in both meat and meat products.
• Lipolysis can be anti- or pro-oxidative depending of various factors.
• Phospholipase A2 inhibits hemoglobin-mediated lipid oxidation in washed muscle.
• Depletion of lipid hydroperoxides is one possible antioxidant mechanism.
• Free fatty acids convert hemoglobin into a low-oxidative form, hemichrome.

## 1 Introduction

**Fig. 1:**
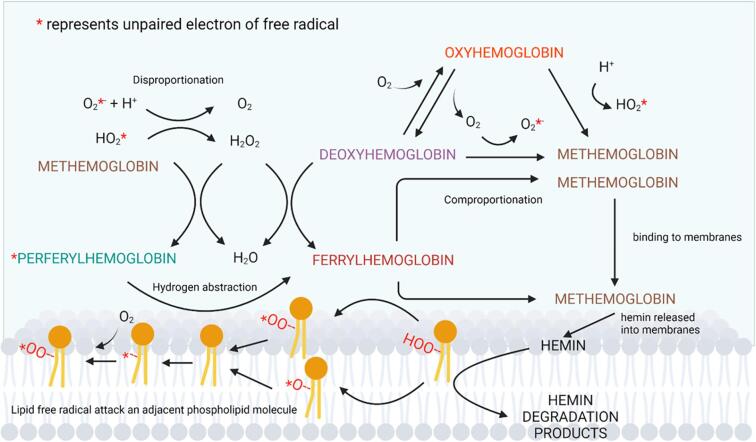
A scheme showing relationship between hemoglobin and lipid oxidation. Oxidation of reduced hemoglobin (oxyhemoglobin and deoxyhemoglobin) results in formations of methemoglobin and superoxide anion, which undergoes spontaneous disproportionation into H_2_O_2_ and O_2_ under acidic conditions. A reaction between methemoglobin and H_2_O_2_ yields hypervalent ferryl hemoglobin radical which can directly abstract hydrogen from lipid molecules to form lipid free radicals. Comproportionation between ferrylhemoglobin and deoxyhemoglobin accelerates the formation of methemoglobin which subsequently binds and releases hemin into the membranes. The hemin mediates decomposition of pre-formed lipid hydroperoxides into alkoxyl radicals that propagate lipid oxidation or form secondary lipid oxidation products via beta-scission.

Lipids are one of the most important components of meat and meat products (Amaral, da Silva, & Lannes, 2018). Meat contains pro-oxidants which are transition metals and heme proteins (i.e., myoglobin and hemoglobin) that can promote oxidation of unsaturated lipids resulting in quality deterioration e.g. discoloration (due to oxidation of heme proteins) and off-aroma (due to generation of volatile compounds) (Richards, 2010). Fig. 1 shows relationship between oxidation and heme proteins (represented by hemoglobin) and lipids, which have been featured in several review articles for example, Maqsood et al., 2012, Wu et al., 2022. Despite that, chemical degradation of lipids play a crucial role in the formation of the final flavor of product and are primarily responsible for both desirable and undesirable flavors and aromas in meat or fish (Huang et al., 2014, Wu et al., 2015). Lipid degradation in meat is mainly due to lipolysis of enzymatic origin (Vestergaard, Schivazappa, & Virgili, 2000) and to chemical oxidation (Wu, Yan, Zhuang, Huang, Zhao, & Zhang, 2016). Hydrolysis is an early step in the conversion from lipids to flavor compounds (Huang et al., 2014) by forming free fatty acids (FFA) from triacylglycerols and phospholipids (Jin, Zhang, Yu, Zhang, Lei, & Wang, 2010). Muscle lipases and phospholipases such as acid lipase, neutral lipase and phospholipase are generally responsible for the lipolysis seen in meat and meat products (Andres, Cava, Martin, Ventanas, & Ruiz, 2005). However, in addition to endogenous lipases, microbial lipases also contribute to lipolysis, especially in dry-cured and fermented meat products (Chen et al., 2017, Xu et al., 2018).

The oxidation of FFAs is the second step in the conversion from lipids to flavor compounds (Huang et al., 2014). Lipid oxidation, including auto-oxidation and enzyme-catalyzed oxidation of fatty acids, causes the formation of hydroperoxides (Jin et al., 2010). Hydroperoxides are degraded into secondary oxidation products such as aliphatic aldehydes, alcohols, ketones and esters by a series of complex reactions. Lipoxygenases make key contributions to lipid oxidation in many different processed meat products (Toldra, 1998, Yang et al., 2018, Wu et al., 2022). A moderate level of lipid oxidation can have a positive effect on the development of the typical flavor of processed meat products (Marušić Radovčić, Poljanec, Petričević, Mora, & Medić, 2021). On the other hand, a high level of lipid oxidation may affect color, texture, nutritional value, taste, and aroma leading to rancidity which is responsible for off-flavors and unacceptable taste of meat and meat products (Amaral, da Silva, & Lannes, 2018). Various studies have shown that free fatty acids produced from lipolysis are the main precursors of volatiles and that volatiles formed as a result of lipid oxidation are responsible for the flavor characteristic of different processed meat products such as dry-cured Iberian ham, cecina, dry-cured loin (Lorenzo, 2014, Pateiro et al., 2015). However, there is a paradox among researchers regarding the relationship between lipolysis and lipid oxidation. While some researchers (Coutron-Gambotti and Gandemer, 1999, Yang et al., 2005) claimed that intense lipolysis could promote lipid oxidation, the others (Gandemer, 2002, Jin et al., 2010) postulated that lipolysis did not have a close relation with lipid oxidation. Therefore, articles dealing with the relationship between lipolysis and lipid oxidation, two important phenomena that occur in meat and meat products and are closely related to product properties, are very substantial. This review focuses paradoxical effects of lipolysis on lipid oxidation in meat and meat products.

## 2 Factors influencing lipolysis in meat and meat products

### 2.1 Targets of lipolysis

Triacylglycerols and phospholipids are an important source of free fatty acids formed as a result of lipolysis (Huang et al., 2014). Gandemer (2002) reported that the relative contribution of these lipids depends on the triacylglycerol content of the raw material. He also declared that triacylglycerols also provide a great amount of FFAs (30–50%) in muscles with high triacylglycerol content. However, there are also studies showing that triacylglycerols remain almost unchanged, while phospholipids contributed to the formation of FFAs (Toldra, 1998, Yang et al., 2005). Yang et al. (2005) stated that phospholipids are the main substrate of lipolysis in the intramuscular lipids of Chinese Xuanwei ham. Similarly, Xu, Xu, Zhou, Wang, and Li (2008) declared that phospholipids, especially phosphatidylethanolamine was the main contributor to the increase of FFAs in a traditional dry-cured meat product. These implications for phospholipids are consistent with the hypotheses that the FFA composition is close to that of phospholipids and the decrease in content of phospholipids in muscle during processing of dry-cured meat products (Coutron-Gambotti & Gandemer, 1999).

### 2.2 Lipolytic enzymes

**Fig. 2:**
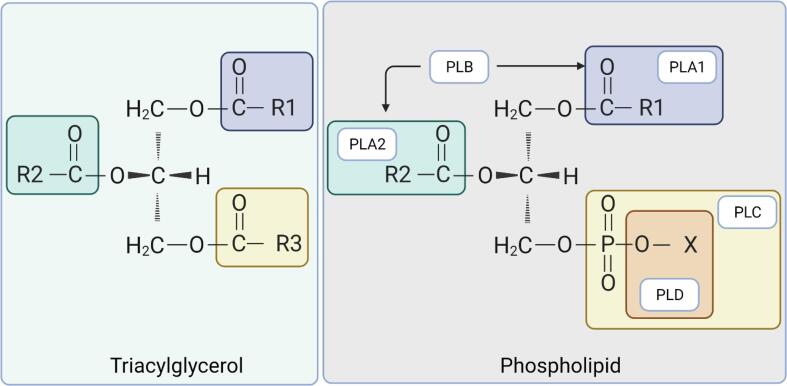
Cleavage sites of lipases and phospholipases on the Fisher projections of a triacylglycerol molecule (left) and on a phospholipid molecule (right). R_1_ R_2_ and R_3_ represent fatty acid molecules at the *sn*-1, *sn*-2, and *sn*-3 positions, respectively. X represents the head group of the phospholipid molecule.

Lipolysis, which leads to the formation of FFAs, is governed by a number of specific enzymes. In this context, both endogenous enzymes of fat cells and muscle fibers and bacterial enzymes play an important role in lipolysis. Lipoprotein lipase and hormone-sensitive lipase systems play an important role in the hydrolysis of triacylglycerols (Gandemer, 2002). However, Toldrá (1998) reported that phospholipases are of great importance in dry-cured ham, since a large percentage of the FFAs formed result from phospholipid hydrolysis. Generally, phospholipases can be classified into 4 classes each designated with a letter: A, B. C, and D, according to their specific cleavage sites on the phospholipid molecule shown in Fig. 2 (Ramrakhiani & Chand, 2011). Phospholipases A1 (PLA1) and A2 (PLA2) selectively de-esterify and liberate fatty acids from the *sn*-1 and *sn*-2 positions, respectively. Meanwhile, an enzyme that exhibits both the PLA1 and PLA2 activities is called phospholipase B (PLB). On the other hand, Phospholipases C (PLC) and D (PLD) show catalytic actions on the phosphodiester bonds at the *sn*-3 position of the phospholipid molecule.

### 2.3 Muscle type

Some researchers reported that different lipolytic activity in muscles might be related to genetic factors (Monin, Hortos, Diaz, Rock, & Garcia-Regueiro, 2003). Toldra and Flores (1998) reported that metabolic muscle type affects the lipid composition and enzyme activity levels, and this causes different flavor development in products. Alasnier, David-Briand, and Gandemer (2000) stated that the metabolic type affected the FFA amount of muscles and oxidative muscles contained freer fatty acid than glycolytic ones due to the high triacylglycerol content of oxidative muscles. They also reported that the rate of phospholipid hydrolysis was close to each other in all investigated muscles. In addition, it has been hypothesized that intrafibre triacylglycerols could be more readily hydrolyzed than triacylglycerols of adipose cells located between fibers. It has been stated that this condition is associated with intrafibre triacylglycerols are an *in situ* reserve of fatty acids quickly mobilized to provide fuel for beta-oxidation to supply energy (Alasnier, David-Briand, & Gandemer, 2000).

### 2.4 Processing conditions

Manufacturing of meat products can affect lipolysis due to processes and ingredients involved; for example, mechanical processes of size reduction and mixing release the lipolytic enzymes and increase access to lipid substrates (Jamilah, Abbas, & Rahman, 2008). The characteristics of meat products and processing conditions such as way of salting and ripening duration vary from one product to another. Therefore, differences are observed in the lipid degradation that takes place during the maturation of the products.

The effects of salts on the progression of lipolysis have been reported in salt-treated meat products (Lorenzo et al., 2015, Mariutti and Bragagnolo, 2017). Vestergaard, Schivazappa, and Virgili (2000) associated the increase in FFA observed during ripening in dry-cured ham with the increase in lipase activity as a result of decreased water activity resulting from progressive dehydration and salt diffusion. Andres et al. (2005) stated that salt have a slight promoting effect on lipolysis at concentrations below 6% in production of Iberian dry-cured ham. Liu et al. (2019) found that the phospholipase activities in dry-cured beef increased after treatment with a salt substitute containing l-Lys and l-His. On the other hand, there are also some researches showing that salt inhibits or slows down lipolysis phenomenon (Jin et al., 2010, Harkouss et al., 2015). Jin et al. (2010) determined that the activities of the lipolytic enzymes gradually decrease during processing stages and this is partly due to the salt content. It should be noted that not all lipases respond to salt similarly. For example, salting above 20 g/L could either inhibit muscle neutral lipases and neutral and acid esterases or activate acid lipases (Toldra & Flores, 1998). Therefore, the lipolytic changes in this type of meat products are attributed to lysosomal acid lipase and hormone-sensitive lipase with optimum pH values of 5.5 and 7.0–7.5, respectively (Vestergaard, Schivazappa, & Virgili, 2000) when the pH value of dry-cured meat products generally varies between 5.5 and 6.2 (Lorenzo, 2014, Gómez et al., 2018, Bou et al., 2022).

The parameters such as time and temperature applied during production stages of meat products are other factors affecting lipolysis. In this context, there are several studies showing an increase in FFA content in the production of dry-cured meat products. Hernandez, Navarro, and Toldra (1999) determined a 10-fold higher level of FFA in a dry-cured meat product compared to the raw material. Alasnier, David-Briand, and Gandemer (2000) investigated the level of lipolysis of rabbit muscles during refrigerated storage and they found that the amount of FFAs increased during refrigerated storage. However, Muriel, Andres, Petron, Antequera, and Ruiz (2007) determined that there was no significant difference in the FFA profile of fresh and dry-cured loin due to the much higher amount of neutral lipid than polar lipid. Andres et al. (2005) declared that use of different temperatures during the drying stage of Iberian dry-cured ham has no effect on the changes affecting the amount of fatty acids of the different lipid fractions.

In fermented meat product, there are conflicts about the contribution of microbial-derived lipolytic enzymes to lipolysis (Xu et al., 2018). Some researchers have reported that the contribution of bacterial lipases to the degree of lipolysis in fermented sausages is very limited and even some selected strains exhibit no lipolytic activity (Kenneally et al., 1998, Lorenzo et al., 2014). The environmental conditions in dry fermented sausages are far from the optimal conditions of bacterial lipases (Gandemer, 2002). Nevertheless, there are also various studies in the literature showing that some cultures such as *S. warnieri*, *S. xylosus* isolated from various processed meat products exhibit high lipolytic activity and that these strains contribute significantly to the level of lipolysis that occurs during fermentation or ripening process (Gao et al., 2016, Chen et al., 2017). Casaburi, Di Monaco, Cavella, Toldra, Ercolini, and Villani (2008) reported that microorganisms with high lipolytic activity can be selected as starter cultures for promote flavor development in short ripened meat products. The differences of microbial lipases on lipolysis may be related to different product, different process conditions and starter culture differences used.

## 3 Relationship between lipolysis and lipid oxidation in meat and meat products

### 3.1 Detection of lipolysis and lipid oxidation

In fresh muscles and in processed meats, several lipolytic enzymes, both endogenous and exogenous, still remain active and are responsible for hydrolysis of lipids even during refrigerated or frozen storage, leading to simultaneous losses of lipid substrates and increases in hydrolysis products e.g. FFAs (Bligh and Scott, 1966, Hardy et al., 1979, Sklan et al., 1983, Brake and Fennema, 1999, Shang et al., 2021). Therefore, FFAs are often used for monitoring the extent of lipolysis due to availability of rapid chemical or enzymatic colorimetric assays (Falholt et al., 1973, Mizuno et al., 1980). Titration for acids in organic solvent extracts are also employed for measuring FFAs (Huang, Xiong, Kong, Huang, & Li, 2013). Sherazi, Mahesar, Bhanger, van de Voort, and Sedman (2007) developed a rapid Fourier transform infrared spectroscopy technique for determination of FFAs that required small amounts of lipid extracts and could be done within 2 min of determination. Spectroscopic and chromatographic techniques i.e., thin-layer chromatography, liquid column chromatography, gas chromatography, and mass spectrometry, can also be employed for profiling liberated FFAs and measuring losses of lipid substrates e.g. triacylglycerols and phospholipids, which give more information as to the sources that contribute to FFA liberation (Bligh and Scott, 1966, Sklan et al., 1983, Kotosai et al., 2013, Balakrishna et al., 2020, Chao et al., 2020). For lipid oxidation, peroxide value which measure lipid hydroperoxides (Feng, Tjia, Zhou, Liu, Fu, & Yang, 2020) and thiobarbituric acid reactive substances (TBARS) which measure lipid oxidation-derived carbonyl compounds, primarily malondialdehyde (Wu, Yin, Zhang, & Richards, 2017), are two commonly reported lipid oxidation indices, although other oxidative indices e.g. formation of carbonyl compounds and loss of sulfhydryl group are also reported along with these two (Wu et al., 2016, Zhao et al., 2019). Table 1 summarizes some of the techniques for determining lipolysis and lipid oxidation.

**Table 1:** Examples of detection techniques of lipolysis and lipid oxidation.

Phenomena	Parameters	Techniques	References
Lipolysis	Lipid substrate content	High performance liquid chromatography	Bligh and Scott, 1966, Hardy et al., 1979, Sklan et al., 1983, Brake and Fennema, 1999, Shang et al., 2021
		Thin layer chromatography	Kotosai et al. (2013)
		Mass spectrometry	Chao et al. (2020)
	Free fatty acids	Colorimetric methods	Falholt et al., 1973, Mizuno et al., 1980
		Titration	Huang et al. (2014)
		Gas chromatography	Monin et al., 2003, Liu et al., 2019
		Fourier transform infrared spectroscopy	Sherazi et al. (2007)
		Mass spectrometry	Chao et al. (2020)
Lipid oxidation	Primary lipid oxidation products (lipid hydroperoxide, peroxide value)	Colorimetric methods	Whalin et al. (2021)
		Spectrophotometry (conjugated dienes and trienes)	Sklan, Tenne, and Budowski (1983)
	Secondary lipid oxidation products	Colorimetric methods (TBARS, malondialdehyde content)	Monin et al., 2003, Chen et al., 2021
		Gas chromatography (hexanal content)	Whalin et al., 2021, Bou et al., 2022
		Electronic nose	Wang et al. (2019)

### 3.2 Lipolysis and lipid oxidation in raw meat

Raw meat is subjected to lipolysis and lipid oxidation during cold and frozen storage; however, the relationship between the two phenomena appears complicated as shown in Table 2.

**Table 2:** Summary of select literatures cited on lipolysis and lipid oxidation in meat and processed meat products.

Authors	Meat and meat product	Condition	Observations
Aberoumand and Baesi (2020)	Fish *Lethrinus atkinsoni* fillet	Frozen storage at −18 °C	Free fatty acid content, peroxide value and TBARS increased with storage time.
Zhang et al. (2020)	Bighead carp (*Hypophthalmichthys nobilis*) fillet	Repeated freezing (−18 °C) and thawing cycles (FT cycles)	Free fatty acid content, peroxide value and TBARS increased with number of FT cycles. Silver carp (Hypophthalmichthys molitrix) fin hydrolysates partially inhibited lipolysis and lipid oxidation.
Castell et al., 1966, Castell and Bishop, 1969	Cod fillet	Frozen storage at −12, −18, and −25 °C	Unlike fresh fillet, frozen cod fillets became resistant to lipid oxidation induced by either Cu2 + or hemoglobin. The resistance to Cu2 + -induced lipid oxidation increased with the free fatty acid content.
Tatiyaborworntham and Richards, 2018, Tatiyaborworntham et al., 2021	Washed cod muscle	Cold storage on ice at 2 °C	Adding phospholipase A2 from porcine pancreas to washed cod led to increase in free fatty acid. Peroxide value, TBARS, and hemoglobin oxidation were inhibited by phospholipase A2 treatment.
Hassanin et al. (2017)	Chicken duck turkey thigh and breast	Short term storage on ice during transfer to laboratory	Free fatty acid content: duck > chicken > turkey Lipid oxidation (peroxide value and TBARS) : duck > chicken > turkey
Alasnier et al. (2000)	Chicken thigh and breast	Cold storage at 4 °C	Thigh contained 3-fold more free fatty acid content than breast. Breast had TBARS 4-fold more TBARS than thigh.
Zhang et al. (2021a)	Cherry Valley duck breast	Cold storage at 4 °C	High cytosolic phospholipase A2 expression was found in low malondialdehyde breasts.
Tatiyaborworntham and Richards (2015)	Chicken leg mince	Cold storage on ice at 2 °C	Adding phospholipase A2 from porcine pancreas to chicken leg mince led to increase in free fatty acid. Peroxide value and TBARS were enhanced by phospholipase A2 treatment.
Chao et al. (2020)	Pork loin *longissimus* muscle	Vacuumed and aged at 4 °C	Phosphatidylcholine was the primary substrate of hydrolysis by phospholipase A2 due to increase in lysophosphatidylcholine content. Increase in phosphatidic acid content suggests phospholipase D activity. Malondialdehyde content was not different during the 21 days of ageing.
Zhao et al. (2020)	Chinese dry sausage with 2% and 4% salt content	Chinese dry sausage process	Lipid oxidation and lipolysis were higher in 4% salt sausage. Higher neutral and acid lipase activities were observed at 4% salt. Phospholipase activity was not affected by salt content.
Chen et al. (2021)	Cherry Valley duck breast	Vacuum tumbling, static brining, and pulsed pressured salting	Static brining and pulsed pressured salting had the higher malondialdehyde content than vacuum tumbling. Vacuum tumbling had more free fatty acid content than the other two method.
Huang et al. (2014)	Chinese traditional smoke-cured bacon	Chinese traditional smoke-cured bacon process	Free fatty acid content increased after salting and smoking steps. Phospholipids were the main contributors of the free fatty acids. Peroxide values and TBARS continued to increase throughout the process. Lipoxygenase activity was highest during salting and the initial stage of smoking. Phospholipase activity declined along the process.
Nachtigall et al. (2019)	Salted bovine *biceps femoris*	NaCl partially replaced by KCl and CaCl_2_ at the same ionic strength Vacuum-packed and stored at 25 °C	The formulation with CaCl_2_ led to higher lipolysis and malondialdehyde content.
Whalin et al. (2021)	Pork sausage treated with phospholipase A2 and rosemary extract	frozen storage at −20 °C	A combination of phospholipase A2 and rosemary extract decreased lipid hydroperoxide content, especially in the neutral lipid fraction. The combination also preserved redness and lowered hexanal content of unseasoned sausage.

#### 3.2.1 Fish

Fish are highly susceptible to lipolysis especially during frozen storage possibly due to integrity of lipid membranes and leakage of hydrolytic enzymes along with required co-factors e.g. calcium ion, causing quality deterioration (Burgaard and Jorgensen, 2011, Standal et al., 2018). In addition, lipolytic enzymes that are able to operate at low temperatures with relatively high catalytic efficiency may be essential for some fish that inhabit in cold environments (Sovik and Rustad, 2005, Kurtovic et al., 2009). In general, lipids from fish are enriched with polyunsaturated fatty acids (PUFAs) such as eicosapentaenoic acid (EPA) and docosahexaenoic acid (DHA) (Wu, Abdollahi, & Undeland, 2021), which are readily oxidized. Concomitant increases in FFAs and lipid oxidation products e.g. TBARS have been observed during frozen storage in several studies (Refsgaard et al., 2000, Aberoumand and Baesi, 2020, Zhang et al., 2020). Refsgaard, Brockhoff, and Jensen (2000) found that thermal inactivation of lipid-hydrolyzing enzymes in salmon mince prevented increases in FFA and lipid peroxide contents during 6 month storage at −10 °C. Comparing between the 3 lipid fractions: neutral lipid, FFA, and polar lipid, the authors also noted that neutral lipids were the main contributors of FFAs, while FFA and polar lipid were the lipid fractions that underwent oxidation in salmon. Repeated freezing and thawing augmented lipolysis and lipid oxidation in bighead carp (Zhang et al., 2020). These observations led to a hypothesis that lipolytic accelerated lipid oxidation due to FFAs being more susceptible to oxidation than the esterified ones. In order to counteract the undesirable effects of lipolysis and lipid oxidation, Mi, Guo, and Li (2016) studied the effect of 6-gingerol, a biologically-active phenolic compound of ginger, at 50 and 100 mg/kg as inhibitors of lipid oxidation and lipolysis in red drum (*Sciaenops ocellatus*) fillet under refrigeration. Similarly, Zhang et al. (2020) developed a strategy of using silver carp (*Hypophthalmichthys molitrix*) fin hydrolysates that can partially retard both FFA release and lipid oxidation in bighead carp fillets that were subject to 6 cycles of freezing-thawing. Wang et al. (2019) used sodium pyrophosphate and sodium tripolyphosphate to control lipid oxidation in tilapia fillets during ice storage. Although the phosphates inhibited oxidation of lipid, the FFA content appeared to be higher in the phosphates-treated fillets than in the control fillets. The authors concluded that oxidation of FFAs in the control samples was responsible for the discrepancy. In contrast, Torres-Arreola, Soto-Valdez, Peralta, Cardenas-Lopez, and Ezquerra-Brauer (2007) explained that lipid oxidation-derived short chain acids contributed to the higher FFA content in sierra fish (*Scomberomorus sierra*) fillets packed in low-density polyethylene films during −25 °C storage and that incorporation of butylated hydroxytoluene into the film reduced both FFA content and lipid oxidation products.

Despite evidence of lipolysis leading to lipid oxidation, studies on certain fish species, in particular cod, have reported that following cycles of freezing and thawing, fish contained a greater amount of FFAs and became less sensitive to lipid oxidation promoted by copper and iron ions (Castell, Moore, Jangaard, & Neal, 1966) and by hemoglobin (Castell & Bishop, 1969). Similar observation were made by Hardy, Mcgill, and Gunstone (1979) who reported that despite high in PUFAs, phospholipids in cod muscles were hydrolyzed by 30% and oxidized very slowly during prolonged storage of 200 days at −10 °C. Due to the majority of lipids in white muscle fish being membranal phospholipids which were the primary targets of hydrolysis in cod (Bligh and Scott, 1966, Hardy et al., 1979) and mackerel (Brake & Fennema, 1999), the antioxidant role of phospholipases thus became of interest. Experimentally, a positive relationship between the amount of FFAs and stability of lipids as indicated by suppressed formation of TBARS was observed in rainbow trout (Mazeaud and Bilinski, 1976, Shewfelt et al., 1981) and in muscle microsomes from flounder (Shewfelt, Mcdonald, & Hultin, 1981).

Washed fish muscles have been used as lipid substrate models for studying mechanisms involved in lipid oxidation (Harrysson et al., 2020, Tatiyaborworntham et al., 2021, Wu et al., 2022). The process of repeated washing removed endogenous water soluble anti-oxidants and pro-oxidants, leaving behind muscle matrix, lipid membranes, and lipid soluble components; therefore, pro- and anti-oxidants of interest can be studied using washed muscle models (Wu, Xiao, Yin, Zhang, & Richards, 2021a). Tatiyaborworntham and Richards (2018) studied the antioxidant effect of PLA2 against trout hemoglobin-mediated lipid oxidation in washed cod muscle. They found that not only did PLA2 inhibit formations of primary and secondary lipid oxidations, but also retarded oxidation of the heme protein. Moreover, the formation of ferrylhemoglobin which is a hypervalent oxidizing form of hemoglobin that was found to be involved in the pro-oxidant effect of trout hemoglobin (Lee, Tatiyaborworntham, Grunwald, & Richards, 2015), was found to be suppressed by the PLA2 treatment. Further study reveals that availability of calcium ion and pH are important factors that govern hydrolytic activity and antioxidant effect of PLA2 against Hb-promoted lipid oxidation (Tatiyaborworntham, Yin, & Richards, 2021).

#### 3.2.2 Poultry

Similar to fish, chicken, duck, and turkey are also subject to lipolysis and lipid oxidation during cold storage (Sklan et al., 1983, Alasnier et al., 2000; Hassanin, A, Elsheikh, & Amin, 2017; Abdullah, Buchtova, & Jezek, 2022). The correlation between lipolysis and lipid oxidation during cold storage seem to be ambiguous in poultry. During −18 °C storage, phospholipids seem to be the primary sources of FFAs in turkey breast and thigh and formation of conjugated oxidation products of lipids appeared to increase as phospholipid content decreased (Sklan, Tenne, & Budowski, 1983). Hassanin et al. (2017) compared inter-species lipolysis and lipid oxidation in chicken, duck, and turkey. In both thigh and breast cuts, the FFA content was in the declining order: duck > chicken > turkey, which is the same for the lipid oxidation indices, which were peroxide value and TBARS. Interestingly, turkey thigh and breast contained higher proportions of PUFAs, namely linoleic acid, linolenic acid, and arachidonic acid, than the other two birds. It should be noted that the elevation of FFAs does not necessarily promote lipid oxidation. For example, Alasnier et al. (2000) reported that during 4 °C storage of chicken, thigh had 3-fold more PUFA FFAs than breast, but up to 4-times less TBARS. Despite that, confounding factors such as vitamin E content may be interfering with the interpretation of the observations.

Phospholipase activity, in particular that of PLA2, appears to be related to development of pale soft exudative (PSE) which is a quality trait in chicken *Pectoralis major m.* which is characterized by rapid pH reduction postmortem leading to inferior water holding capacity and color appearance. Soares et al. (2003) found that upon exposure to heat stress, chicken with PSE traits had 1.25 times greater PLA2 activity than non-heat stressed chicken. In addition, the enzyme activity appeared to increase as the animals aged. A combination of lower pH and elevated PLA2 activity was thought to enhance lipid oxidation in PSE chicken (Alessandra de Avila Souza, Shimokomaki, Nascimento Terra, & Petracci, 2022). Despite that, Santos et al. (2012) reported secreted PLA2 activity was found to be similar between PSE and normal breast chicken samples despite higher lipid oxidation in the former. Recently, using an isobaric tag for relative and absolute quantification-based proteomic analysis, Zhang, Wang, Xu, Xu, and Zhou (2021a) found that following 3-day refrigeration, duck breast with lower malondialdehyde content had higher expression of cytosolic PLA2, implying the antioxidant role of the enzyme. Studying the effect of lipolysis products on lipid oxidation, Wu, Xiao, Yin, Zhang, and Richards (2021b) found that 0.05% of mixed FFAs extracted from turkey breast muscle could inhibit hemoglobin-mediated lipid oxidation of ground turkey thigh, possibly due to FFA turning the heme protein into a less reactive low-spin state, hemichrome. However, the pro-oxidant activity of PLA2 has been reported in chicken leg mince during ice storage (Tatiyaborworntham & Richards, 2015).

#### 3.2.3 Mammalians

Large mammalian animals offer a variety of meat cuts which show diverse sensitivity to lipolysis and lipid oxidation. For example, during −10 °C storage, pork belly cut had lower FFA but higher TBARS than loins (Park et al., 2007). The metabolic type dependency was also observed in porcine muscles. Comparing between porcine *Masseter*, *Longissimus dorsi* and *Serratus ventralis* muscles, only the oxidative *Masseter* muscle was found to have significantly greater phospholipid hydrolysis and lipid oxidation (TBARS and hexanal) after 10 days at 4 °C (Morcuende, Estevez, Ruiz, & Cava, 2003). Alasnier, David-Briand, and Gandemer (2000) found that in rabbit muscles, both phospholipids and triacylglycerols contributed to increases in FFAs but the extent depended on the metabolic types of muscles. While the phospholipid hydrolysis was similar among both oxidative and glycolytic muscles, triacylglycerols were slightly preferentially hydrolyzed in glycolytic muscles than in oxidative ones**.** Pre-slaughter treatments can also affect intramuscular lipolysis and lipid oxidation. For example, long fasting time and low glycemic index of feed led to up-regulation of lipolysis-related LIPE gene expression, leading to greater FFA content and, subsequently higher TBARS contents in pork during 8-day storage at 4 °C (Rey, Menoyo, Segura, Lopez-Bote, & Calvo, 2020).

Effect of enzymatic lipolysis on lipid oxidation appears to depend on type of lipolytic enzymes, with phospholipase being antioxidant and lipase pro-oxidant in beef homogenate (Govindarajan, Hultin, & Kotula, 1977). However, considering PLA2, there is still no consensus on the role of the enzyme on lipid oxidation. Comparing *Longissimus lumborum* between Large White and Pietrain pigs, Monin et al. (2003) found that although FFA contents were higher in Pietrain during 9-day vacuum chilled storage, there was no difference in terms of lipid oxidation. In addition, sarcoplasmic and mitochondrial PLA2 activities were not affected by the genetic types, both showing a decrease of sarcoplasmic PLA2 at Day 9 of storage. Similar to PSE in chicken, PSE pork also had higher total and calcium-independent PLA2 activity and TBARS content than normal pork (Chen, Zhou, Xu, Zhao, & Li, 2010). Furthermore, Chen et al. (2010) differentiated between the activity of secretory PLA2 calcium-dependent PLA2, and calcium-independent PLA2 in PSE pork, and proposed that the calcium-independent enzyme had a key role in the development of PSE symptoms. Directly adding PLA2 into pork semimembranosus mince inhibited lipid oxidation (lipid hydroperoxide and TBARS); however, the antioxidant effect of PLA2 was partially diminished by the presence of NaCl (Tatiyaborworntham & Richards, 2015).

### 3.3 Lipolysis and lipid oxidation in processed meats

Lipolysis and lipid oxidation takes place and plays an important role in development of color and flavor characteristics of certain meat products such as aged meats, fermented meats, and cured meats (Lorenzo, 2014, Zhang et al., 2021, Bou et al., 2022). Process procedures can affect lipolysis in meat products. For example, the process of size reduction of meat not only facilitates releases of hydrolytic enzymes and cofactors e.g. calcium ion, from their compartments but also increases access and surface area of substrates that enzymes can catalyze phospholipid hydrolysis. Even after the washing process of preparing surimi, endogenous hydrolytic enzyme activities, namely acid lipase, neutral lipase, and phospholipase still persisted and remained active in the product, and further repeated freeze-thawing led to gradual declines of these enzyme activities (Shang et al., 2021).

Lipolysis occurs during meat ageing, a process of preparing meat cuts for consumption by allowing endogenous enzymes to breakdown connective tissues, tenderizing the meat. Pork loins undergoing ageing showed extensive hydrolysis of phospholipids, primarily phosphatidylcholine, within 8 days, without a significant increase in malondialdehyde, a secondary lipid oxidation product, during 21 day ageing (Chao et al., 2020). In-bag dry-ageing of lambs in water-permeable bags led to about 2.5 times more TBARS than in-bag wet-ageing despite higher FFA content and lower pH in the latter, suggesting lipolysis may not contribute to lipid oxidation (Zhang et al., 2021b).

Fermentation relies on both endogenous and exogenous hydrolytic enzymes to break down macromolecules in meat into smaller substrates that are further metabolized by microbes into flavor and taste compounds. Different formulations of fermented sausages can affect lipolysis and lipid oxidation during maturation of the products. Zanardi, Ghidini, Battaglia, and Chizzolini (2004) tested 4 formulations of Mediterranean and North Europe type fermented sausages by varying the antioxidants ascorbic acid, nitrites and spices in the formulae and found that the extent of lipid oxidation differed among the formulae tested but the lipolysis were unaffected by the recipe changes. Cao et al. (2018) monitored lipid oxidation and activities of lipases and phospholipases of traditional high salt and low salt lactic acid-fermented fish products. All the lipolytic enzyme activities measured declined progressively throughout the process which coincided with increase in lipoxygenase activity and formations lipid hydroperoxide and TBARS. However, principal component analyses did not reveal inversed correlation between lipid oxidation and enzymatically lipolytic activities. Microbial enzymes also contribute to lipid oxidation and flavor development. Chen et al. (2017) reported that inoculation with a starter culture mixture could promote lipid hydrolysis and improve flavor development of Harbin dry sausage. Considering lipid oxidation, TBARS was suppressed significantly in all fermented sausages with single culture, but the best result was obtained from mixed cocktails of the starter cultures. The authors suggested bacterial enzymes e.g. catalase being responsible for the antioxidant effect, which makes it is difficult to directly correlate lipid oxidation and lipolysis.

In salted and cured meat products, lipolysis and lipid oxidation are also of concerns. There are strong positive correlations (R^2^ > 0.75) between TBARS values with enzymatic activities of neutral lipase, phospholipase and lipoxygenase in Chinese dry sausage (Zhao et al., 2020). Curing methods also have a great impact on lipolytic and oxidative quality of cured duck breast. Among vacuum tumbling, static brining, and pulsed pressured salting, vacuum tumbling led to the highest lipolysis but the least lipid oxidation and volatile compounds e.g. 1-hexanol and 1-octen-3-ol, which are identified as off-flavor aroma contributors; therefore, the method can improve aromatic characteristic of the final products (Chen, Luo, Lou, Wang, Yang, & Shen, 2021). Huang et al. (2014) reported that formations of lipid hydroperoxides and TBARS in Chinese traditional smoke-cured bacon were more related to phospholipid hydrolysis than to lipoxygenase activity due to formation of free PUFAs. The phospholipase activity remained relatively constant, contributing to phospholipid degradation and FFA release during the curing process, but declined significantly during the smoking step. There have been attempts made in order to reduce and/or replace NaCl in cured products due to health concern of overconsumption of NaCl (Liu et al., 2019, Nachtigall et al., 2019, Zhao et al., 2020). Substitution of NaCl by CaCl_2_ at the same ionic strength enhanced lipid oxidation and lipolysis in salted meat which altered total volatile profile and shelf life of the products (Nachtigall et al., 2019). Similar observations where partial replacements of NaCl by MgCl_2_ and CaCl_2_ promoted slightly greater lipase activity in dry-cured ham and rapid oxidation of lipids at initial stages of ripening when compared with the 100% NaCl formulation (Ripolles, Campagnol, Armenteros, Aristoy, & Toldra, 2011).

Based on the antioxidant effect of PLA2 in minced pork model (Tatiyaborworntham & Richards, 2015), incorporation of the enzyme into a pork-based product has been tested for its effect on oxidation. Whalin, Liu, Rankin, Zhang, and Richards (2021) reported the antioxidative effect of a mixture between PLA2 and rosemary extract in pork sausage during 245 days of −20 °C storage. The authors measured lipid hydroperoxides in 3 lipid fractions: neutral lipid, FFA, and polar lipid, and found that lipid hydroperoxides in the neutral lipid fraction of pork sausage treated with PLA2 and rosemary extract decreased by 3.3-fold. Similar observations on the effect of PLA2 on reduction of lipid hydroperoxides were previously reported in pre-oxidized washed cod muscle (Tatiyaborworntham & Richards, 2018).

## 4 Possible mechanisms involved in lipolysis-mediated lipid oxidation in meat

### 4.1 Lipolytic products

Regarding effect of lipolytic products on lipid oxidation, FFAs received most attention, while only few studies were focused on lyso-phospholipids (Szebeni & Toth, 1986). In general, liberated fatty acids, especially highly unsaturated ones, become more vulnerable to oxidation than their esterified counterparts, although it was not always the case (Shen et al., 2021). Under certain conditions, non-esterified PUFAs may act as free radical scavengers in human aortic endothelial cell (Richard, Kefi, Barbe, Bausero, & Visioli, 2008) and stimulated macrophage (Ambrozova, Pekarova, & Lojek, 2010) models. Mazeaud and Bilinski (1976) proposed that free PUFAs might act like free radical scavenger, and upon oxidation, undergo cyclic rearrangement to form prostaglandin instead of breaking down into secondary lipid oxidation product.

Being amphiphilic in nature, non-esterified fatty acids are free to orient themselves at the interface between the hydrophilic surroundings and the hydrophobic part of lipid structures e.g. membranes or droplets, attracting pro-oxidant metals into close proximity of lipids (Waraho, McClements, & Decker, 2011). Alternatively, at critical micelle concentrations, FFAs may diffuse into aqueous medium to form spontaneous aggregates, known as micelles, increasing the surface area which can lead to greater exposure to oxygen and pro-oxidants. Moreover, FFAs become substrates for lipoxygenases, a group of iron-containing oxidoreductase enzymes, which catalyze dioxygenation of fatty acids with region-specificity, a phenomenon that can be pro-oxidant in postmortem muscles and processed meats (Fu et al., 2009, Yang et al., 2018, Liu et al., 2019). Given its hydrolytic activity, PLA2 has been shown to be involved in lipoxygenase-mediated oxidation of fatty acids to form lipid hydroperoxides and superoxides in skeletal muscles (Zuo, Christofi, Wright, Bao, & Clanton, 2004). However, oxidation of FFA at the *sn*-2 position of phospholipids by lipoxygenase may as well take place before the hydrolysis by PLA2 (Chaitidis, Schewe, Sutherland, Kühn, & Nigam, 1998), which is aligned with the preference of PLA2 towards oxidized phospholipids (Litvinko, Skorostetskaya, & Gerlovsky, 2018). The uses of phosphatidylcholine-binding protein and PLA2 antibody were tested to alleviate the negative impacts of phospholipid hydrolysis (Wang et al., 2014, Chun et al., 2022).

The types of lipolytic enzymes and fatty acids seem to influence the oxidation of lipids. A study using beef homogenates as lipid substrates indicated that lipolysis by phospholipases was antioxidative, whereas lipases exacerbate lipid oxidation in meat models (Govindarajan, Hultin, & Kotula, 1977). Phospholipids contain relatively higher PUFAs, especially at the *sn*-2 position which is the target site for PLA2, than triacylglycerols do (Dominguez, Pateiro, Gagaoua, Barba, Zhang, & Lorenzo, 2019) and liberation of these fatty acids was studied for their influence on lipid oxidation. Mixed FFAs from seal bubbler and cod liver oil provide protection to cod mince against Cu^2+^-induced lipid oxidation, while pure linoleic or linolenic acids did not show any antioxidant effect (Castell et al., 1966). In oil-in-water emulsion models, oleic acid was the most potent pro-oxidant followed by linoleic and linolenic acids, respectively (Waraho, McClements, & Decker, 2011), which is contradict to the idea that higher the unsaturation of fatty acids, lesser the stability. The authors attributed the greater pro-oxidant effect of oleic acid partially to the abilities of the fatty acid to migrate to the water–lipid surface and attract pro-oxidative metal ions due to more reduction in the surface charge (zeta-potential) of lipid droplets.

Related to lipid oxidation in muscle matrices, FFAs were found to exacerbate oxidation of oxymyoglobin into metmyoglobin, a form of myoglobin with peroxidase-like activity that can promote decomposition of peroxides and propagate lipid oxidation (Stewart, 1990). However, evidence is presented that FFAs may exert antioxidant effects by neutralizing heme proteins, which are major pro-oxidants in meat. Abilities of FFAs to bind and induce conformational change of heme proteins (myoglobin, hemoglobin) into hemichrome, which is a more inert form of the pro-oxidants, have been demonstrated (Stewart, 1990, Wu et al., 2021b). Litvinko, Skorostetskaya, and Gerlovsky (2018) determined PLA2 activity by monitoring the conversion of hemoglobin into hemichrome upon reacting with FFAs released from the enzymatic hydrolysis of phosphatidylcholine liposomes and micelles.

### 4.2 Membrane remodeling

Lipid membranes are highly vulnerable to oxidation due to degree of unsaturation and large surface area with aqueous surroundings that deliver oxygen and pro-oxidants into contact (Dominguez et al., 2019). As aforementioned, iron and heme proteins are crucial pro-oxidants in meat and meat products and the crucial first step of heme protein-promoted lipid oxidation is likely mediated by electrostatic interactions. The negatively charged polar head groups of phospholipids attract the pro-oxidants to the surface of lipid membranes (Shviro et al., 1982, Sannaveerappa et al., 2014). For heme proteins, hydrophobic interactions induce structural changes, leading to dissociation of the heme moiety which intercalate itself into the bilayers (Shviro et al., 1982, Szebeni et al., 1988, Mahato et al., 2012). In washed cod muscle model, binding of heme and hemoglobin is associated with lipid oxidation due to interactions between pre-formed lipid hydroperoxides and the heme compounds (Sannaveerappa et al., 2014, Tatiyaborworntham et al., 2021). Therefore, it was hypothesized that preventing interactions between pro-oxidants and their lipid substrates may prevent oxidation.

**Fig. 3:**
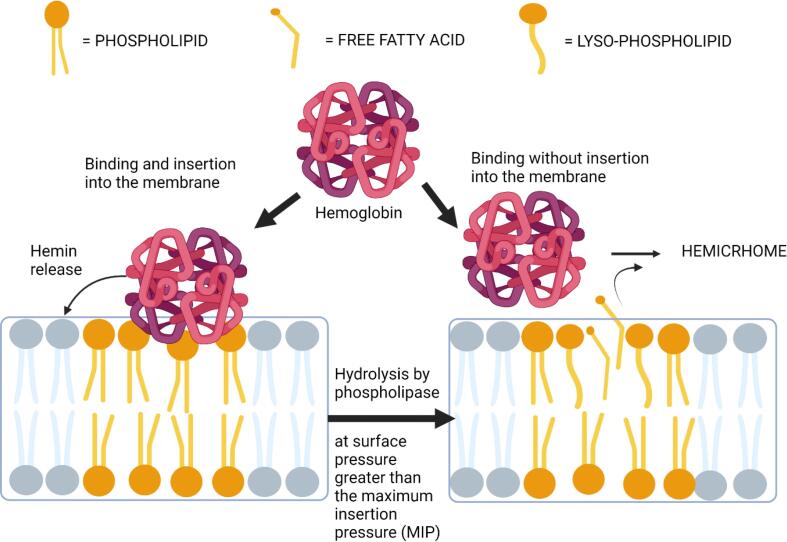
Interaction between hemoglobin with lipid membranes and the effect of phospholipase. Hemoglobin binds and releases hemin into the fluid lipid membranes. Hydrolysis of phospholipids by phospholipase yields fatty acids and lyso-phospholipids which occupy more lateral space within the lipid layers, increasing the surface pressure as the result. At the maximum insertion pressure (MIP), hemoglobin may bind to the membrane surface but penetration into the membrane is prohibited. In addition, binding of the released fatty acid may convert hemoglobin into hemichrome which is an inert low-spin form of hemoglobin.

Hydrolysis activity of PLA2 has roles in remodeling lipid membranes (Leidy, Ocampo, Duelund, Mouritsen, Jorgensen, & Peters, 2011) and may affect lipid oxidation preventing molecular insertion of hemin into lipid membranes which is governed by fluidity and lipid surface pressure of lipid membranes (Szebeni et al., 1988, Mahato et al., 2012). Enzymatic hydrolysis splits phospholipids into FFAs and lyso-phospholipids, some of which remain in the membranes. The increasing numbers of molecules crowding within the membranes creates a rise in surface pressure, preventing molecular insertion into the membrane as shown in Fig. 3 (Mahato et al., 2012). However, pre-treating washed cod muscles with porcine PLA2 did not seem to prevent deposition of hemin into the insoluble fraction of the washed muscle which consisted of lipid membranes and myofibrillar proteins (Tatiyaborworntham, Yin, & Richards, 2021). Despite that, PLA2 was able to inhibit hemin-mediated lipid oxidation regardless of the hemin binding to the insoluble fraction of the washed muscle.

Hydrolysis of lipid membranes can induce segregation of membranal lipid constituents into lipid domains, i.e. fluid liquid-crystalline phase and rigid gel phases (Leidy et al., 2011). Aggregation of PUFA residues of phospholipids in the fluid phase means an localized increased concentration of oxidizable unsaturated lipids and facilitation of lipid radical propagation (Schnitzer, Pinchuk, & Lichtenberg, 2007). PLA2 can promote an increase of the gel phase in a mixed 1,2-dimyristoyl-*sn*-*glycero*-3-phosphocholine (DMPC)/1,2 distearoyl-*sn*-*glycero*-3-phosphocholine (DSPC) system, due to hydrolysis of DMPC and enrichment of DSPC (Leidy et al., 2011). However, due to lack of sufficient supporting evidence, it is not clear how hydrolysis of membrane lipids can change the dynamics of membrane properties in regard to lipid oxidation.

### 4.3 Removal of preformed lipid hydroperoxides

**Fig. 4:**
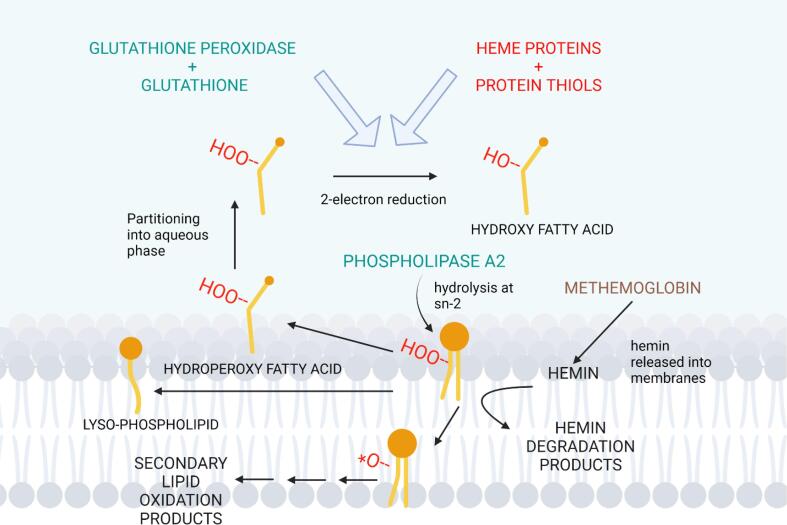
Phospholipase A2 interferes with hemin-mediated decomposition of pre-formed lipid hydroperoxides into alkoxy radicals. The formed lipid radicals can either attack neighboring phospholipid molecules or undergo beta-scission into secondary lipid oxidation products e.g. malondialdehyde. Phospholipase A2 hydrolyzes and releases hydroperoxy fatty acids from the *sn*-2 position of oxidized phospholipid molecules. The hydroperoxy fatty acids may partition into the aqueous phase where they are reduced to hydroxyl fatty acids by glutathione peroxidase or heme proteins. Glutathione and protein thiols serve as reducing entities that provide electrons for converting the proteins into their active forms.

Presence of preformed lipid hydroperoxides enhances susceptibility of lipid oxidation by hemoglobin (Szebeni and Toth, 1986, Richards and Li, 2004). In the presence of heme compounds, lipid hydroperoxides can be decomposed into lipid radicals and hypervalent heme species that can initiate further lipid oxidation reactions (Wu, Richards, & Undeland, 2022). Therefore, preferential hydrolysis towards oxidized phospholipids due to presence of membrane defects (Litvinko, Skorostetskaya, & Gerlovsky, 2018) could be the role of PLA2 in depleting the membranes of pre-formed lipid hydroperoxides as illustrated in Fig. 4. Crude enzyme extracts from chicken, pork, and beef muscles possess phospholipase activity, with PLA2 being around 45–49% of all the hydrolytic activity, towards oxidized phosphatidylcholines, yielding hydroperoxy fatty acids as hydrolysis products (Balakrishna et al., 2020). Tatiyaborworntham and Richards (2018) found that porcine PLA2 was able to inhibit progressing lipid oxidation by adding the enzyme to washed cod muscle at different stages of undergoing hemoglobin-mediated lipid oxidation: lag phase, exponential phase and end phase. Regardless of the stage of lipid oxidation, the lipid hydroperoxides ceased to increase when the enzyme was added, while TBARS slightly increased. These observations suggest that PLA2 could interfere with the progression of lipid oxidation, in addition to ridding membranes of pre-formed lipid hydroperoxides.

Glutathione peroxidase and glutathione reductase are involved in the conversion of the liberated hydroperoxy fatty acids into hydroxyl fatty acids, a lesser pro-oxidative form (Tan, Meyer, Belin, & Ketterer, 1984). In addition, Kotosai et al. (2013) also suggested a similar role of high-density lipoprotein (HDL) in detoxification of hydroperoxy fatty acids liberated from oxidized low-protein lipoprotein by platelet-activating factor-acetylhydrolase. By using Chloramine-T, a methionine-specific oxidant, the results revealed that the methionine residues of apolipoprotein A-1 in HDL was responsible for the 2-electron reduction of 13-hydroperoxy octadecadienoic acid into 13-hydroxyoctadecaenoic acid. Peroxiridoxin 6 which is a bifunctional cytosolic enzyme with 2 active sites for glutathione peroxidase and acidic, calcium-independent PLA2 activities, which allows the enzyme to directly reduce oxidized esterified fatty acids and liberate the fatty acids from the phospholipid molecule (Fisher, 2011). However, while the peroxidase prefers neutral cytoplasmic pH, the hydrolytic activity is most active at acidic pH of lysosomes, suggesting that these two activities may work independently with the former being involved in getting rid of oxidized fatty acids from membrane and the latter in metabolism of phospholipids.

In addition to antioxidant, tocopherols also have roles as membrane stabilizers by forming complexes with FFAs and lyso-phospholipids upon phospholipolysis (Wang & Quinn, 2000), which was hypothesized that this might recruit tocopherols into close proximity of the released hydroperoxy fatty acids by PLA2 (Tatiyaborworntham, Yin, & Richards, 2021).

## 5 Conclusion

The roles of lipolysis on lipid oxidation remain inconclusive since information obtained from most reported studies only suggests correlation, rather than causal relationship, between the two parameters. Several intrinsic factors such as lipid substrates, lipolytic enzyme activity, and presence of anti- and pro-oxidants can influence the outcome. Furthermore, presence of microbial starter culture in fermented sausage further complicates the interpretation of the results. However, under controlled experimental conditions, phospholipases have been demonstrated to provide antioxidative protection against lipid oxidation, especially when pro-oxidants are heme proteins. Hypotheses of the antioxidant mechanism of PLA2 are based on the preferential hydrolysis of PLA2 towards oxidized phospholipids. Since hemin/heme proteins reactively break down pre-formed lipid hydroperoxides in a bimolecular fashion to form lipid radicals, propagating the lipid oxidation reaction cascade, clearance of the lipid hydroperoxides from membranes removes one of the two reactants from the equation. Alternatively, changes in the physical properties of membranes induced by PLA2 may interfere with how hemin/heme proteins interact with the membranes, which prevents the interaction between the pro-oxidants and the lipid hydroperoxides. Studying the mechanisms behind the antioxidant effect of PLA2 can be crucial for protection of meat and meat products from undesirable effects of lipid oxidation.

## Declaration of Competing Interest

The authors declare that they have no known competing financial interests or personal relationships that could have appeared to influence the work reported in this paper.
